# Monthly Summary

**Published:** 1867-10

**Authors:** 


### MONTHLY SUMMARY.

---

*Chemistry of the Secretions.*—Dr. J. H. Salisbury, Professor of Histology, Physiology and Cell Pathology in Charity Hospital Medical College of Cleveland, Ohio, has been making a series of researches, which he has published in the *St. Louis Medical Reporter.* We extract from the paper in that Journal the following summary of results.—(*Eds. Amer. Journal.*)

The following appear to be the main facts elicited from the foregoing experiments, and from some brief concluding remarks relating to other experiments and investigations, which are too extended to be introduced in this paper:

1. The salivary secretions excite the panary fermentation in wheat dough.

2. The salivary secretions excite, also, in moistened wheat dough that has been raised by them, the developement torula (yeast) cells, quite as abundantly and readily as brewers' yeast, and, consequently, must excite in farinaceous matter alcoholic fermentation.

3. The salivary secretions excite the acetous and putrefactive fermentations in wheat dough.

4. The salivary secretions do not excite any perceptible alcoholic fermentation in solutions of sugar, but slowly develope acetous and putrefactive changes.

5. The salivary secretions hasten putrefactive fermentation in fresh meats.

6. Torula cells have been noticed in the blood of the living body, indicating the presence of sugar, undergoing fermentative changes.

7. Algoid and fungoid spores occur in the blood of fevers, in that of erysipelatous inflammations, and in that of diabetes.

8. The cell products of parent cells have a tendency to take on filamentous developement, and those filaments make up much of the basement structure of the animal body.

9. It appears that one important office of the lymphatics is, to more perfectly assimilate, transform and organize albuminous matters into fibrine, and that they are not exciting organs, but strictly organs of assimilation.

10. Cholesterine occurs in the vitreous and aqueous humors and crystalline lens of the eye.

11. Seroline occurs as a secretion in, and an excretion of, the prostate gland.

12. Certain forms of the lithates of ammonia and soda of Golding Bird are deposited in developing cells that emanate from the epithelial tissue of the urinary surfaces.

13. Cholesterine and seroline are formed quite largely by the spleen. This organ appears to be one of the principle sources of the cholesterine and seroline that are formed in the animal body.

14. The pancreas, in the hog, appears to possess the power of transforming cholesterine into seroline.

15. The thyroid gland of the hog contains seroline.

16. Peyer's glandulæ and the mucous follicles of the large intestines, and the lower portion of the small intestines, eliminate seroline, mixed with cholesterine in the last stages of transformation into seroline.

17. The cholesterine, secreted from the blood by the liver and poured into the alimentary canal as a constituent of the bile, appears not to be an excretion proper of that organ, as is evident from the fact that it is mingled with the chyme (as a constituent of the bile) and aids in transforming this substance into chyle, with which it is taken up by the lacteal system, and, during the elaboration of the nutrient materials in their passage through the lacteal glands, becomes partially transformed into seroline. This renders it probable that cholesterine, with other products of the bile, perform some useful function in the assimilation and *organization of the nutrient materials.*

18. Cholesterine is not eliminated from the body as such to any great extent, but is transformed usually first into seroline, before it *becomes an effete product.*

19. Cholesterine is taken up with albuminous matter from the blood in its capillary circulation by the lymphatics, and, during its passage through the lymphatic glands, appears to become partially transformed in seroline.

20. The Cholesterine of the food of the bile appears to be partially transformed into seroline by the lacteal glands. These glands also appear to have the power, to some extent, of forming cholesterine in the process of organizing fibrin cells from the albuminous materials of the chyle.

21. Epithelial cells are extra-vascular, and appear to be organisms analogous to cells of the zoosporoid type, possessing a vitality independent of the animal to which they are attached, and placed between the dead world and the living animal tissues; their function being to feed upon crude matters, and so change their character in their transit through them as to fit them to act as food for the more highly animalized parent cells inside the basement membrane.

22. That the epithelial cells covering the intestinal villi appear to be the primary agents in the taking up and organization of the chylous materials of the food; that they fit a portion of the materials transmitted through them to be appropriated by the capillary vessels; while the balance passes on to be taken up by the lacteals, and to be organized by the parent cells of the lacteal glands.

23. That much of what is called endosmotic and exosmotic action appears to arise primarily from the vitality of living cells; and, as such is not a property of dead matter, but one of living cells, by which living cell organisms are fed and nourished. It is simply a process of cell-feeding.

24. That the important and primary function of the lacteal and lymphatic glands is to organize fibrin cells ; from which, by metamorphic changes, a portion of filamentous fibrin and blood discs of the blood are formed ; also, a portion of the nerve products, including cholesterine and seroline.

25. That the important function of the spleen is to organize fibrin, blood discs, and cholesterine and seroline, and some other nerve products.

26. That it is the primary function of the lobules of the liver to organize fibrin cells and animal glucose ; and that of the interlobular cells and biliary glandules to form the biliary secretions.

27. The biliary glandules terminate the biliary tubuli in the interlobular spaces.

28. A primary function of the kidneys and supra-renal capsules is to form fatty substance, and perhaps bony matters, and a peculiar class of fibrin cells, resembling somewhat those of the liver ; and that the secretion of urine appears to be a resultant process of the organization of these products.

29. *That the primary form of fibrin, when first organized from albuminous matters by the parent cells of the glands, is a cell* enclosing (in mammalia) a large central nucleus, surrounded by many minute cells. That, subsequently, this cell, save its cell contents, is "spun" or metamorphosed into a filament, which filament is a secondary form of fibrin, the form of blood fibrin, and in which condition it is fitted to permeate the thin-walled capillaries, and be appropriated by the living fibrinous tissues.

### CONCLUDING REMARKS.

Cholesterine and seroline are formed quite largely in the spleen during the organization of cells (fibrin cells and blood discs) in this organ. The cell products of the spleen have been treated of fully in a paper on this subject, published in the American Journal of Medical Sciences, April, 1866. The ultimate structure and functions of this gland are highly interesting, and explain many obscure and long sought for physiological processes. There is strong evidence for believing that this gland instead of the brain, as has been supposed, is one of the principal sources of the cholesterine that originates in the animal body. Unless the brain be admitted to be in part a gland, we ought to have suspected that it necessarily could have itself no power to organize the ultimate products of which it is composed. It is the peculiar function of glandular structures in their varied arrangement to organize the ultimate basement products of which the tissues of the body are built up and sustained. The tissues, save the fibrinous, cartilaginous and bony, have simply the power of appropriating materials organized for them by glandular structures. There is, however, some probability, from the presence in the nervous system of nucleated cells, which appear analogous to parent gland cells, that it may perform glandular functions, but of what extent is not known.

There is also evidence for believing that the lacteal and lymphatic glands form cholesterine and seroline to some considerable extent during the performance of their function of organizing fibrin cells, &c., from the albuminous materials of the chyle. There is also evidence of these glands performing the office of transforming, in some degree, the cholesterine of the chyle (nutrient products of the food mixed with bile) into seroline.

Further, there is evidence that the bile is a product of the interlobular, epithelial cells, from which it is secreted by *biliary glandulæ*, which are found to terminate the biliary tubuli in the interlobular spaces, It appears, although an excretion of a certain class of hepatic cells, not to be an excretion proper of the liver, but a secretion, secreted by the biliary glandulæ for specific purposes in digestion and assimilation. The evidences of this will be fully set forth in another paper on the ultimate structure and functions of the liver.

---

*Absorption of Medicines.—The Southern Journal of the Medical Sciences*, for August, contains a translation of a paper by M. Demarquay on this interesting subject, of the general results of which we make a brief abstract.

We all know that absorption from the gastro-intestinal surface is constantly relied on, but the rate at which that absorption goes on, and its relative activity, as compared with other mucous, serous and dermal surfaces, has not been carefully and scientifically studied. Mr. Demarquay has attempted to supply this deficiency. The substance he selected was iodide of potassium, on account of its known rapidity of absorption and facility of recognition. The secretions in which evidences of absorption were sought, were the urine and the saliva. Sometimes the one and sometimes the other of these secretions manifested the presence of the medicines with greater rapidity. As a general thing, evidences of the presence of the salt can be obtained from these secretions in from 9 to 15 minutes after its introduction into the stomach.

Different opinions have been entertained by practical men as to the relative rapidity of absorption by the upper and lower tracts of the intestinal canal. It has been customary, for example, to give much larger doses of medicinal agents by the rectum than by the mouth. Dupuytren, however, maintained that absorption of opium was more rapid in the rectum than in the stomach. The experiments of our author confirm the opinion of the great surgeon. He obtained evidence of the presence of iodine in the blood, from 2 to 7 minutes after the introduction of iodide of potassium into the rectum as an enema.

The mucous membrane of the bladder absorbs feebly. The time required to detect iodine in the saliva after the injection of a large quantity of iodide of potassium into the bladder, varied from 35 minutes to 6 hours. In one half of the observations none whatever was recognized.

The mucous membrane of the glans and prepuce in the male and of the vagina in the female, absorb but feebly in the natural state; when diseased, however, ulcerated or abraded, absorption in these regions is very rapid.

The bronchial mucous membrane is very prompt and rapid as an absorbing surface. When iodide of potassium is inhaled in the form of spray from an atomizer, it makes its appearance in the urine in 5 or 6 minutes.

Serous membranes absorb readily. Injections into the tunica vaginalis of the testicle, passed into the circulation, in times varying from 7 to 38 minutes.

The question of absorption by the skin is rather more intricate. It is well known that when the surface is painted with tincture of iodine, the urine speedily gives indications of the presence of iodine. Unfortunately, however, for the hypothesis of cutaneous absorption, the surgeon and the bystanders are found to have iodine in their urine also. The absorption has taken place not through the skin but through the mucous membrane of the air passages. Experiments with baths gave our author usually negative results. Out of sixteen baths containing a large quantity of iodide of potassium, (nearly a quarter of a pound in most of them) no evidence of absorption was discoverable in ten. In eight a feeble indication of iodine was found in the urine. The author thinks these even doubtful as proving absorption from the general cutaneous surface, as the genito-urinary mucous membrane might have been credited with the result. To determine more accurately the activity of the skin, he resorted to inunction. An unguent made of pure lard and iodide of potassium was applied to the leg and thigh of a patient, which was then carefully enveloped with gum taffeta, wadding and bandages. In twenty experiments of this kind, a small quantity of the salt was detected in the urine. Dry iodide is never absorbed.

Perhaps the author was not sufficiently persistent in his use of the iodide in baths. Hoffmann took every third day, for six weeks, a bath containing 770 grains of iodide potassium. After the fifth bath only did that distinguished chemist recognize the presence of the iodide in his urine. It is remarkable that elimination in this case was as slow as absorption, since the salt could be detected in the urine twelve days after the baths had been discontinued.

---

*Mixed Chloroform and Ether.*—Robert Ellis, in a communication to the London Medical Times and Gazette, points out several objections to the common practice of mixing these two anæsthetics.

First—The ingredients of these mixtures evaporate to a great extent independently of each other and in very different quantities.

Second—That though alcohol exerts *in vacuo* a powerful influence in equalizing evaporation, this influence is almost lost in evaporation in free air.

Third—To procure a true anæsthetic mixture, due attention must be paid to the relative volatility of the ingredients, which must be mixed in such proportions as to insure their simultaneous evaporation.

Fourth—The quantity of ether required to insure this result is so great that the mixture becomes equivalent to ether alone.

Fifth—If impure alcohol and ether be used, these defects are exaggerated and the result is vitiated by the water left behind after evaporation.

A mixture of one part of chloroform and two of ether, commonly employed in England, was experimented upon. Half a drachm was poured over a definite evaporating surface, and in a hundred seconds nearly all the ether had evaporated. For three minutes the evaporation of chloroform went on, and in four minutes all had gone, only a faint odour of alcohol remaining.

The same mixture was distilled at a gentle heat, not exceeding 120°. Before distillation, its specific gravity was 990. At the end of half an hour, the distillate had a specific gravity of 910. At the same time the density of the residue in the retort was 1120, showing that the ether had evaporated much more rapidly than the chloroform. Thus the ether, which is relied upon to stimulate and so counteract the depressing action of the chloroform, is used up at the beginning, and the latter anæsthetic is left to itself, at the most critical period of the anæsthesia.

As a substitute for this dangerous and untrustworthy compound, Mr. Ellis reccommends the use of the "mixed vapours." He has contrived an apparatus by which the three different vapours can be inhaled either separately, or combined, in such proportions as the operator may choose.

---

*Ozone.*—The Rev. Samuel Haughten, M.D., F.R.S. has been writing on the meterogical causes of Asiatic Cholera. In these papers he attacks the notion so long prevalent that ozone destroys the cholera poison. He maintains, first, that ozone is a constant or nearly constant quantity in the atmosphere, and that the different tints of the test papers are due to the greater or less prevalence of winds which bring unequal quantities of air to act upon the iodide. He cites, in proof of this, the experiments of Mr. Smyth, who placed ozone in a tube connected with an aspiratur, thus securing a regular flow of air. He found that the tint was the same every day, while exposed papers were subject to the greatest variations in colour. The curves of the anemometer were also found to correspond closely with those of the ozonometer. Secondly, he claims that there is no demonstrable proportion between cholera and ozone. The curves of the two certainly show no correspondence; on the contrary, the very day that the cholera was most fatal, ozone was in greatest abundance.

